# S50. Proffered paper: Complex tumour microenvironment screening platform captures biological responses of cancer therapeutics

**DOI:** 10.1186/2051-1426-2-S2-I12

**Published:** 2014-03-12

**Authors:** H Layman, J Melrose, W Tang, N Brown, D Nguyen, A Melton, M Polokoff, E Berg, A O'Mahony

**Affiliations:** 1 DiscoverX, Biology, South San Francisco, CA, USA; 2 DiscoverX, HTS Robotics, South San Francisco, CA, USA

## 

The tumour microenvironment consists of a heterogeneous population of cancer-associated fibroblasts, leaky vasculature, polarised and suppressed immune cells, and hyperproliferative, transformed and invasive epithelial cells. Capturing the complex phenotypic biology of the tumour microenvironment *in vitro* is extremely challenging as numerous components drive a malignant phenotype. Currently, cancer drug screening is limited by lengthy clinical trials, rodent models unable to capture human biology, and *in vitro* screening in monoculture which ignores complex cell interactions. An *in vitro* co-culture platform to mirror these cancer microenvironments may permit phenotypic screening of multiple cancer drugs to elucidate additive or synergistic effects prior to clinical trials.

We have recently developed a panel of primary human co-cultures, BioMAP^®^ Oncology Systems, which mimic the complexity of host-tumour stromal and vasculature microenvironment. By using primary human fibroblast or endothelial cells with stimulated peripheral blood mononuclear cells (PBMCs) and the human epithelial adenocarcinoma cell line HT-29, we are able to successfully model a stromal or vascular tumour microenvironment. These co-culture models allow us to generate a unique profile of biological responses for drugs tested at multiple concentrations. These biological responses are a function of 41 cell-based and soluble protein readouts, measured by immune-based methods, examining tumour-associated immunomodulation, angiogenesis, matrix remodeling, and cellular proliferation. Using paclitaxel as a model cancer drug we found that it exhibited immunostimulatory characteristics through upregulation of soluble GranzymeB, TNFα, and IFNγ. Upregulation of these readouts is found in the clinic consistent with cancer cell death via T-cell-dependent anti-tumour effects. We also screened typical standards of care (SOC) for colon cancer, gemcitabine and carboplatin, to determine their response profiles. Alternatively, these systems can be used to screen potential cancer drugs in combination or matrix format to assess therapeutic strategies for synergistic or additive effects. Profiling of cancer immunotherapeutics (e.g ipilimumab) in combination with current SOC provides insightful data for clinicians to understand immune-preserving and cancer-cell killing activities.

Profiling of compounds across a panel of tumour microenvironment co-cultures provides a powerful experimental platform in primary human cell-based system that allows researchers in early compound development to investigate their drug’s potential therapeutic targets, and, at late-stage development, accelerate a drug(s) track through clinical testing. The ever increasing number of clinical trials using multiple cancer drugs can be reduced by utilising a matrix of drugs administered in our newly developed BioMAP^®^ oncology systems to determine which drugs in combination are of clinical efficacy.

